# A Scoping Review on the Effects of COVID-19 on Syringe Service Programs in the United States

**DOI:** 10.7759/cureus.39023

**Published:** 2023-05-15

**Authors:** Dylan Pietrantoni, Crystal Barroca, Sarah Lynch, Jonathan Byrne, Miranda Ortner, Roshni Kotwani, Kolin Limbrick, Paul Kaldas, Michael Moussa, Tatem Fredrickson, Jeffrey Schaefer, Robin J Jacobs

**Affiliations:** 1 Dr. Kiran C. Patel College of Osteopathic Medicine, Nova Southeastern University, Fort Lauderdale, USA

**Keywords:** community health research, syringe, pwid, persons who inject drugs, ssp, syringe service program, pandemic, covid-19

## Abstract

The COVID-19 pandemic has had worldwide impacts, including disrupting community services. One interrupted service was syringe service programs (SSPs), community-established initiatives that provide sterile supplies and aid in overcoming addiction in drug-using participants. In the United States (U.S.), SSPs have been key in combating the recent opioid use crisis and associated infections such as the human immunodeficiency virus (HIV) and hepatitis C. While some published reports on the pandemic’s overall impacts on SSPs exist, certain aspects such as operational changes and repercussions on staff and participants may still be lacking. Information about the impact of interrupted SSP services due to the pandemic may provide insight into how to prepare to mitigate similar outcomes during possible future health outbreaks. The aim of this scoping review was thus to explore the effects of the COVID-19 pandemic on the operations, staff, and participants of SSPs in the U.S. The initial search of the databases PubMed, Embase, and Web of Science with selected keywords yielded 117 articles published in English between January 1, 2020, and August 31, 2022. After screening each article for study eligibility, 11 articles were included in the final review. Of the seven articles exploring SSP operational impacts from the pandemic, five acknowledged that mitigation strategies influenced functions, seven highlighted supply changes, and four emphasized the resulting staffing changes. Four studies inspected the pandemic’s impacts on SSP participants, which included two articles highlighting participants’ struggles with isolation and loneliness, one referencing the fear of exposure to the SARS-CoV-2 virus, and two examining the overall negative psychological effects experienced during this time. SSPs in various settings and regions across the U.S. experienced changes due to the COVID-19 pandemic. Many of these modifications negatively impacted operations, staffing, and participant relationships. Examining the issues that individual SSPs encountered highlights opportunities for structured solutions for the present and in the case of future infectious disease outbreaks. With the severity of the opioid use crisis in the U.S. and the dependence on SSPs for its mitigation, future work in this space should be prioritized.

## Introduction and background

Since the COVID-19 pandemic began, most public services such as government agencies and healthcare facilities were suspended while the properties of the virus and how the illness developed were being studied [1]. The "lockdown" from the pandemic had severe impacts due to the suspension of essential services. According to a survey conducted by the World Health Organization (WHO), 23% of countries worldwide stated that the pandemic had disrupted at least 75% of healthcare services offered, with lower socioeconomic groups reporting a considerably higher percentage of halted services [2]. Some of these services, such as syringe service programs (SSPs), include initiatives aimed at halting the spread of human immunodeficiency virus (HIV) and hepatitis and combating the current drug crisis in the United States (U.S.).

The U.S. is currently in the midst of an opioid crisis due to the misuse of opioid painkiller prescriptions, along with heroin and fentanyl [3]. The rise in substance use disorders (SUD) has resulted in an increase in overdose deaths and hepatitis infections, as well as hazardous working environments for medical personnel when needles are not disposed of properly [3]. In the U.S., SSPs were established to provide a variety of services to persons who inject drugs (PWID) to negate some of these adverse outcomes [3].

SSPs are community-based initiatives that aim to improve the health of PWID. Introduced in the 1980s, SSPs primarily allow PWID to dispose of used material and access sterile needles in order to reduce transmission of bloodborne illnesses [4]. Additionally, SSPs connect PWID with substance abuse treatment programs, provide immunization and testing for common bloodborne pathogens, and offer education about safe drug use [4]. Because many locations now provide naloxone for treatment, SSPs have also decreased overdose deaths [5]. SSPs are widely regarded by public health experts as safe, effective, and cost-conscious models that mitigate harm for PWID [6]. Understanding how SSPs work and how they assist the local community during a pandemic can be advantageous in the event of future pandemics.

Although SSPs have played an important role in reducing bloodborne infection rates in PWID since the 1990s, there has been a recent increase in injection drug use and, along with it, outbreaks of hepatitis and HIV [4]. The Centers for Disease Control and Prevention (CDC) asserts that the majority of new hepatitis C infections are due to injection drug use and that new HIV infections associated with injection drug use have substantially increased in the mid-2010s [4,7,8]. In tandem, there has been a surge in opioid-related overdose deaths in the U.S. since 2013 due to the widespread availability of intravenous drugs, like fentanyl, heroin, and other synthetic opioids [9,10].

Another factor now influencing the rise in drug-related morbidity and mortality is the COVID-19 pandemic. Declared a worldwide pandemic by the WHO in March 2020, COVID-19 has been shown to affect certain populations, such as older adults, individuals with certain medical conditions, and PWID [11] disproportionately. During the height of the pandemic, researchers noted a large reduction in screening rates for HIV and hepatitis, resulting in a poor ability to track data on new infections [12,13]. Similarly, due to shutdowns, many at-risk individuals were no longer able to obtain access to Pre-Exposure Prophylaxis (PrEP), a regimen to reduce the risk of acquiring HIV [14]. Finally, opioid overdose deaths surged during the pandemic. Researchers hypothesize that this is due to a decline in mental health due to isolation, economic distress from the pandemic, changes to the drug supply chain causing unsafe mixtures of drugs, and lack of access to substance use treatment programs [15-17].

Moreover, safety measures taken during the COVID-19 pandemic resulted in any non-essential healthcare services shutting down, disproportionately affecting marginalized communities, such as PWID [18]. Socioeconomic factors predispose PWID to harmful effects from COVID-19; these include stigma causing reluctance to seek care, financial insecurity leading to inability to obtain personal protective equipment (PPE), a high prevalence of homelessness and lack of safe housing leading to crowded, unsafe living conditions, and communal drug use [19]. Fortunately, the CDC released a statement in May 2020 declaring SSPs to be an essential service that they recommended staying open during the COVID-19 pandemic to help combat these negative outcomes.

Prior research on SSPs, however, has indicated that because SSPs vary widely (i.e., state vs. local, urban vs. rural, mobile vs. fixed locations), there may be wide discrepancies in how they are operated and the challenges they face, such as community attitudes towards the programs [16,20]. This could mean that, despite CDC guidance, individual SSPs may have approached operations differently during the pandemic due to varying population characteristics between locations [21-24].

Currently, little is known about the ways SSPs changed their models during the COVID-19 pandemic. Additionally, SSPs have historically been funded by city, county, and state health departments, and community-based organizations. The effects of the pandemic have impacted SSP funding, and it is uncertain what degree of funding will return to SSPs as pandemic restrictions are now removed [25]. Further studies are also needed to help underline the differences in how COVID-19 affected SSPs in rural versus urban settings [26]. Lastly, it is unclear how individual SSP policy changes were decided and what the consequential impacts were.

Understanding the ways in which SSPs adapted to the pandemic and altered their roles in the PWID community is vital for the current and possible future outbreaks. While some literature has been published on the influence of COVID-19 on SSPs, certain aspects, such as demographic discrepancies, operational changes, impacts on SSP staff, community attitudes, and individual approaches by SSPs, have not been reviewed. The purpose of this review was to further elucidate the role of SSPs during the COVID-19 pandemic and clarify the impact of COVID-19 on SSPs in the U.S. A further understanding of the COVID-19 pandemic’s impacts may help re-evaluate methods for providing SSPs, which have become important in addressing ongoing public health concerns during the nationwide pandemic generated lockdowns.

## Review

Methods

The method for this scoping review was based on the framework of Arksey and O’Malley that includes five stages for conducting systemized reviews: (1) identifying the broad review question; (2) identifying studies in a comprehensive manner; (3) selecting relevant studies that meet the inclusion and exclusion criteria; (4) data recording; and (5) collating, summarizing, and reporting the results [27].

Eligibility Criteria

Two tiers of the review were used to assess if articles fit the inclusion criteria. Article inclusion criteria were based on the target patient population, the study design, and the study concept. To meet the inclusion criteria, articles had to be peer-reviewed, written in English, and published between January 1, 2020, and August 31, 2022. Articles were included if they evaluated the effects of COVID-19 on the SSPs and if the study populations included those who use SSPs ('participants') or those that operate ('operators') or staff ('staff') SSPs within the U.S. Quantitative, qualitative, and mixed-method studies were included.

Search Strategy

The Population, Concept, and Context (PCC) strategy was utilized for structuring the research question, "How has the COVID-19 pandemic impacted syringe exchange programs across the United States?" The population included SSPs in the U.S. The concept broadly included the impacts of the COVID-19 pandemic on those programs, and the context was defined as the era of the pandemic in the U.S. ending in August 2022 for the purpose of data collection. The research team agreed upon the terms for the search, and one author conducted the searches independently based on those terms before aggregating the sources for all authors’ reviews. Searches across all databases included synonyms and terminological variations with Boolean operators to broaden the results. The initial search evaluated titles and abstracts that included the following terms: "COVID-19", "SARS-COV-2", or "coronavirus" and "syringe" or "needle" and "service", "program", or "sharing".

Information Sources

**Figure 1:**
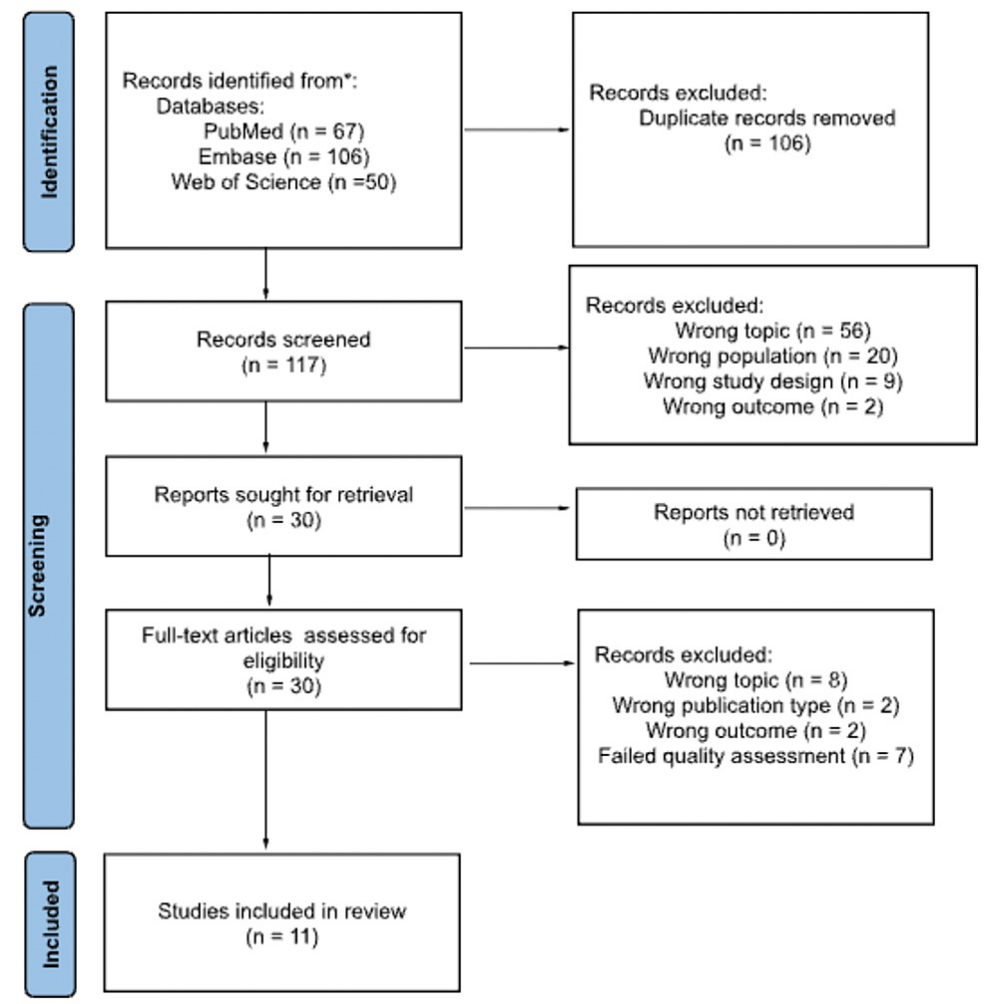
PRISMA Flow Chart of the Article Screening and Selection Process PRISMA: Preferred Reporting Items for Systematic Reviews and Meta-Analyses

The research team collectively agreed upon the inclusion criteria, search terms, and databases to be used in the paper. The initial search used the following databases: PubMed, Embase, and Web of Science. Initial data collection took place on October 8, 2022. The search found 223 sources. First, 106 duplicates were removed, leaving 117 sources for screening. Research team members then worked individually to evaluate the titles and abstracts for relevance to the review. Seven articles were unanimously included, 58 were unanimously excluded, and 52 had mixed initial evaluations. The secondary review team assessed those 52 articles and decided 23 articles met the inclusion while 29 did not. Of all 87 articles excluded in the first-tier review, the reasons for exclusion were 56 for the wrong topic, 20 for the wrong population, nine for the wrong study design, and two for the wrong outcome. After the assessment of the remaining 30 articles by the second-tier review team, 19 articles were excluded: eight for wrong topics, two for wrong publication types, two for wrong outcomes, and seven for the failure of the quality assessment [28]. In total, 11 articles were retained for this scoping review. This screening and selection process is illustrated by a Preferred Reporting Items for Systematic Reviews and Meta-Analyses (PRISMA) flowchart (Figure 1).

Selection of Sources of Evidence

All team members discussed the results and inclusion criteria to set a consistent base before beginning the review of the 117 publications from the initial search; 11 members then worked individually to evaluate the titles and abstracts of the publications to determine the relevance to the study. The secondary review team resolved any discrepancies in article selection after the 11 members’ reviews.

Data Charting Process

The data-charting process was started using Excel. Three reviewers extracted and charted the information from the sources on the form. Each reviewer independently determined the relevant details to include. Any inconsistencies in the data were resolved by evaluating and discussing the results to reach a conclusion.

Data Items

Data were extracted from the articles based on the following: data collection formats, population(s) studied, sample sizes, number of SSP sites evaluated, location of the sites (i.e. rural vs. urban), timeframes relative to the outbreak of COVID-19 (i.e., conducted in 2020 vs. conducted in 2021), effects of the pandemic on SSP operations, staff, and patients, and study limitations.

Results

General Description of Selected Studies

Of the 11 studies included in our review, six were semi-structured interviews, and the remaining five included each of the following: mixed-method (semi-structured qualitative interview and survey, in-depth interview, cross-sectional survey, and correlational analysis) [21,23,25,26,29-35]. Four qualitative studies consisted of multiple modalities for data collection (i.e., videoconferencing, telephone) [26,30-32]. Six of those studies allowed at least the option of telephone communication, and five studies allowed at least the option of a videoconference (i.e. Zoom modality) [23,25,26,30-35]. Only two of the articles were published in 2021, and the remaining nine articles were published in 2022 [21,23,25,26,29-35]. However, nine of these studies completed their data collection periods by the end of December 2021 [21,23,25,26,29,31-33,35]. Eight of the 11 studies included SSP programs with more than one site, and the remaining three studies included only one site or did not include this information [21,23,25,26,29-35]. With respect to the locations of the SSPs, three studies reported at least one rural or suburban site, while the other eight did not have a rural or suburban site or did not include this information [21,23,25,26,29-35]. Alternatively, five studies reported at least one urban site, while the other six did not include an urban site or did not include this information [21,23,25,26,29-35].

Summary of the Evidence

Articles were summarized in a table format based on the study design, patient population, findings, and conclusions (Table 1).

**Table 1:** Summary Table of the 11 Articles in the Review

Reference	Study Aim	Study Design	Data Collection Format	Population and Sample Size	Key Findings			Limitations
					Effects on Syringe Service Program Organizations	Effects on Syringe Service Program Operators	Effects on Syringe Service Program Patients	
Aponte-Melendez (2021) [21]	Investigate the negative effects of COVID-19 on persons who inject drugs (PWID) and syringe service programs	Cross-sectional analysis	Telephone, in-person	Participants (N=106)	Placing dispensing machines of harm-reduction supplies in communities where persons who inject drugs live, increasing mobile services, and mailing supplies can help PWID during the COVID-19 pandemic.		Reported decrease in mental health, increase in syringe reuse, and alcohol consumption.	Questions not designed to evaluate the effects of COVID-19; sample was limited to PWID who were positive for hepatitis C virus in New York, NY.
Thakarar (2022) [23]	Identify changes to how harm reduction, treatment and basic medical services were provided in Maine for syringe service programs during the COVID-19 pandemic	Semi-structured interview	Telephone	Staff and participants n=18 participants, n=18 staff	Mobile outreach expansion, mail delivery of equipment, and telemedicine visits were utilized by syringe service programs in Maine during the COVID-19 pandemic.	Staff shortages and exposure risk led to a decrease in availability and supply distribution for their clients. Providers reported an increased demand for supplies as patients were using more drugs to cope with stress.	Increased substance use due to stress and isolation of the pandemic. Inability to access the clean supplies that they needed led to an increase in overdose and transmission.	Telephone-based interviews due to COVID-19 restrictions may have affected responses; demographic data were not assessed, limiting the support for consistency across races and ethnicities
Wenger (2021) [25]	Examine the social context of syringe service program operations during the COVID-19 pandemic	In-depth interview	Videoconference	Staff (N=36)	Introduced secondary and mailing methods for distribution and telemedicine avenues for client enrollment; led to staff shortages due to precautionary measures; reduced current and future funding for operations; reduced operating hours; increased naloxone distributions at each participant visit.	Limited or completely subtracted wages to staff, forcing layoffs or volunteerism, instead of paid work; voluntary resignations due to personal/familial health concerns; decreased interpersonal connections with program patients.		Small sample sizes may limit transferability; self-reporting data are subject to bias.
Glick (2022) [26]	Investigate the effects of the COVID-19 pandemic on syringe service programs in rural Kentucky	Semi-structured interview	Videoconference, telephone, audio recording	Staff (N=18)	Inhibited program expansion and process momentum; paused mobile syringe service program services; introduced virtual meetings for clients.	Deterred the ability to create relationships with the clients, yielding lesser services utilization; further limited time with participating clients due to mitigation precautions.		Findings may not reflect urban establishments; data were collected early in the pandemic, prior to vaccine release.
Allen (2022) [29]	Determine the impact of naloxone vending machines on opioid overdoses	Correlational analysis	District death certificate data	Participants (N=270)	Increased ability to reduce deaths from opioid overdose.	Improved outcomes for patients and potentially less work.	Decreased overdose fatality rates via easy access to naloxone.	Data do not account for mitigation strategy implementation; usage and populations may vary between each site
Austin (2022) [30]	Describe the effects of COVID-19 on syringe service program operations in the United States one year after the pandemic onset	Mixed method (semi-structured interview and survey)	Semi-structured interview: videoconference, telephone Survey: electronic	Staff (N=27)	Forced changes in supply distribution models; created multi-level barriers to syringe exchanges; decreased blood-borne infection testing; introduced positive and negative outcomes of telehealth modalities	Led to disconnect between staff and program participants		Results may not be generalizable due to a small sample size and qualitative methods; data were collected early in the pandemic (February - April 2021)
Feder (2022) [31]	Explore the factors for avoidance of opioid use disorder medications and syringe service programs by people who use drugs in the first year of the COVID-19 pandemic	Correlational analysis	Telephone, online form	Participants N=304 for SSP questionnaire; n=702 for methadone treatment questionnaire			Avoided syringe service programs mostly due to worry about COVID-19	Participants who did not complete the COVID-19 supplemental questionnaire were not included in the study; data represent major metropolitan areas only.
Frost (2022) [32]	Describe how syringe service programs responded to the COVID-19 pandemic and the increasing demand for support to prevent the rise in overdoses which can be attributed to the pandemic	Semi-structured interview	Videoconference, telephone	Staff (N=31)	Most syringe service programs rapidly adapted to the COVID-19 pandemic. COVID related precautions were set such as social distancing, use of PPE, sanitization supplies, and COVID-19 screening.	syringe service programs were placed in a difficult situation as they had to protect their staff, but also try to reach out to help.	The pandemic may be indirectly leading to an increase in transmission of drug use related infections	Data were collected early in the pandemic; responses may have varied due to study modality of videoconferencing versus telephone
Harris (2022) [33]	Analyze the effects of the pandemic on participants with history opioid use disorder seeking addiction services	Semi-structured interview	Telephone	Participants (N=20)			Stratified attitudes about the operational changes ranging from "liberating" to "destabilizing" to "unjust"; Exacerbated feelings of freedom in some participants and isolation in others, with common responses dependent on housing situations and transportation access	Sample is from one geographic location and is narrow in racial differences; category descriptions were devised by the research group, not the participants
Kelly (2022) [34]	Understand the COVID-19 mitigation strategies of syringe service programs and the perceptions of and effects on the syringe service program operators	Semi-structured interview	Videoconference	Staff (N=16)	Reduced operations from 5 to 3 days per week; introduced capacity restrictions in buildings	Created mental anguish while navigating the new operations; loaded more burden onto fully on-site staff, as opposed to off-site counterparts		Findings may not be transferable to other syringe service programs, especially rural and international facilities; response variability may have been influenced by the eligibility for staff to receive COVID-19 vaccine during the research timeframe
Wang (2022) [35]	Evaluate the health and experiences of syringe service program operators during COVID-19 for future emergency response events	Semi-structured interview	Videoconference	Staff (N=18)	Necessitated new COVID-19 policies for ensuring employee physical and mental wellness	Increased anxiety about COVID-19 transmission led to staff turnover; Increased staff burnout from elevated workload		Interviews were only conducted at one point in the early months of the pandemic and lacks longitudinal support; no specific mental health outcomes were evaluated; syringe service programs studied may not be representative of other syringe service programs outside of Massachusetts, which has a strong support from state government

Operational Impacts of the Pandemic

Seven of the 11 studies examined operational aspects of the SSPs, including staffing effects, with responses from SSP staff or administrators [23,25,26,30,32,34,35]. Articles were evaluated for the reference of "mitigation strategies", or contact precautions due to the COVID-19 virus, as outlined in our methods section. Of the seven articles considering the operational effects of the pandemic, five studies acknowledged "mitigation strategies", specifically, playing a role in the changes [23,25,26,32,34]. The reference to mitigation strategies varied across the articles. Based on their findings regarding mitigation strategies, another article claimed that relationships between SSP operators and participants were strained [26]. Another study further elaborated on these strategies, describing new processes for supply pick-ups, outdoor workflows, physical distancing barriers, and mobile delivery services [32].

Supply Changes

Of the seven articles pertaining to the operational impacts of the pandemic, all seven studies referred to supply barriers [23,25,26,30,32,34,35]. The context of these notions ranged across the articles. For example, one study stated that a cascade of complications from the pandemic led to changes in the SSP’s syringe supplier. This barrier caused a limit on the number of syringes and a change to a poorer quality brand of those syringes, which made it more difficult for participants to use [30]. Interviewees in another study explained that they witnessed shortages in safe injection supplies such as sterile cotton and alcohol wipes, and this may have played a role in the increased use of contaminated supplies by participants [23]. Another concern regarding supplies was the reallocation of already limited funding to PPE which put further stress on operations [35].

Staffing Changes

Of the seven articles about the operations of the sites, four mentioned employment changes [23,25,32,35]. These changes included voluntary termination by the staff themselves and involuntary decisions from the administrators. One article emphasized the strain that one SSP faced due to temporarily or permanently reducing staff (i.e. furloughs and terminations, respectively) [25]. Another article mentioned that voluntary staff turnover, possibly due to safety concerns with the virus, quickly cut their employee roster to half-capacity [35]. In fact, independent of the four studies mentioning staffing changes, four of the seven aforementioned articles cited staff having fear or distress over possible exposure to the virus [23,25,34,35]. The aforementioned article also highlighted that remaining staff members were redeployed to other tasks, such as SARS-CoV-2 testing, outside of normal operations, which further stressed the operating system and the staff’s mental health [35]. Notably, staff burnout was mentioned with the changes in staffing by two of the four studies referencing staffing changes and by three of the seven operationally related studies overall [23,25,32,34,35].

Pandemic Impacts on Participants

Isolation and loneliness: Four of the 11 studies included perspectives or analyses of the pandemic’s effects on the participants of the SSPs [21,23,31,33]. Of those four studies, two noted isolation or loneliness having an impact on the lives of the participants during this time [26,33]. One of the articles reported that the isolation from stay-at-home orders during the pandemic disrupted participants’ substance use goals [33]. The other study referenced how changes in SSP operations and implementation of public health policies left participants feeling abandoned and short of resources [23].

Fear of exposure to COVID-19: Of the four studies using participant data, one referenced the participants worrying about the COVID-19 virus and its transmission. This study found a significant association between the self-reported fear around COVID-19 and the risk of avoiding SSPs (OR 3.64, 95% CI 1.66-7.76) [31].

Mental health: Studies were also evaluated for mental health changes or psychological stress on participants due to the pandemic’s impact. Two articles examining participant data noted psychological interruptions during this period [21,33]. One of these studies reported 80.4% of responding participants admitted psychological or emotional problems during COVID-19, as opposed to the 50.0% pre-pandemic (p < 0.01) [21]. Further, the study showed an increase from 52.5% to 84.8% of participants reporting depression symptoms pre- and intra-pandemic, respectively (p < 0.01). Another study described how disrupted supportive community networks (due to the pandemic’s effects) led to escalated substance abuse [33].

Discussion

A total of 11 studies were included in the review and were evaluated for four main purposes: to evaluate the effect of COVID-19 on SSP operations, SSP staffing, isolation, and mental health changes in SSP participants. Respectively, seven, seven, two, and four studies were used for these purposes and are further discussed in this section.

Operational Effects on SSPs

The pandemic forced many companies that supply the necessary equipment to SSPs to halt services or close permanently. One of the studies discussed how the shortage of syringes and safe injection supplies during the COVID-19 pandemic forced SSPs to prioritize certain services over others. For instance, programs ensured the importance of injection equipment for naloxone leading to decreased HIV and hepatitis C virus (HCV) testing. Consequently, staff was diverted to respond to a concurrent HCV outbreak in PWID [26]. Programs also struggled to obtain PPE such as sanitizers and masks, making it harder for PWID to come to in-person testing and injections, further increasing HIV and HCV outbreaks [36]. Moreover, the pandemic increased drug overdose due to clients receiving drugs from unfamiliar suppliers and psychological stressors. The SSPs were unable to combat the rise in drug overdoses coming at a time of limited supply of clean syringes and supplies [36].

Additionally, there was a rise in the rates of solitary drug use by clients due to the isolation constraints caused by the pandemic. SSPs were not capable of consistently supplying clean syringes for solitary use, causing clients to reuse some syringes. Such actions were also potential causes of increased drug overdose and outbreaks of needle-transmitted diseases [26].

Staffing Effects on SSPs

In addition to supply issues, the uncertainty and need for social isolation that revolved around the growing pandemic led to major shifts in the workforce, including the employees for healthcare systems, such as SSPs. This caused major staffing shortages leading to reduced services available. Several studies revealed that the implementation of staff change models addressing fluctuating staff volumes may prove effective in mitigating the reduced services in the future. A study among nursing staff across 25 Texas Health hospital systems explained how using their new staffing models, named "ADKAR" and "CLARC", shifted the focus from individual nursing practices to a team-based approach [37]. In these models, the staff was managed as a continuous team in which effective communication, delegation, and individual mindset allowed for the teams to work together to cover areas with reduced staffing.

Another study examined shortages in staffing and PPE in nursing homes across the country [38]. This study found that over 20% of nursing homes suffered from inadequate supplies of PPE throughout the pandemic. They also found that increased PPE shortages were associated with increased numbers of COVID-19 cases, as well as subsequent shortages in staffing. Addressing the shortages in PPE supplies may play a crucial role in future pandemics and in mitigating the staffing shortages that may occur.

Isolation Effects on SSPs

Aside from more calculable effects on SSP staffing and supplies, the COVID-19 pandemic also had significant social impacts on users, namely increasing feelings of isolation. One of the studies presented in the results section explored the role COVID-19 had in access and agency to addiction treatment and use [33]. Through its interview format, the study found that most participants claimed that the COVID-19 changes to addiction management were either "destabilizing" or "unjust". Many of these participants noted the disruption of routine, abrupt halt to the social interaction provided by community SSPs, and consequences of homelessness on access to take-home methadone policies as drivers to increased substance use [33].

Another study focused on how specific policy changes within cities affected access to harm reduction services [23]. This study used a semi-structured interview to acquire information from community partners and healthcare workers involved in SSPs. Many participants underlined how the shift from face-to-face to more impersonal, isolated forms of communication made users less likely to use SSPs. Participants saw not having face-to-face as a lack of consistency and a lack of physical presence to build that baseline trust, which in turn caused gaps in services [23]. More practically, limited access to cellphones and the internet served as a barrier for many users wanting to use telemedicine. This limited not only access to treatment but also to social interaction and a sense of community.

Overall, social isolation and loneliness have been shown to increase all-cause mortality, including mental and physical health consequences [39]. With addiction falling under the realm of both health issue categories, there is ample evidence pointing to the role of pandemic-related isolation in worsening substance abuse for users. Further, another study investigating the role of social interaction on opioid and benzodiazepine use found that being unmarried led to a three-time increased risk of abusing both substances [40]. Thus, social interaction appears to have a significant impact on drug misuse alone.

Mental Health and Fear of COVID-19 Exposure

Going together with isolation effects on the SSP participants, fear of the SARS-CoV-2 virus also stifled several participants’ progress with addiction treatment, as they avoided picking up medications for treatment during the early months of the pandemic [31]. Even though the pandemic has more recently been waning, this fear has continued to be an excuse for PWID to delay starting treatment.

Furthermore, SSPs played a significant role in participants’ social lives pre-pandemic and acted as a way to socially integrate PWID; shutting down SSPs during the pandemic had a significant negative impact on the mental health of PWID, leading to increases in anxiety, depression, and suicidal thoughts [21]. This decline in community services and increase in mental health struggles may have led to individuals’ relapses. These mental health issues may be the longest-lasting of all pandemic’s effects on SSPs and PWID, as they caused major setbacks for recovery processes and completely disconnected PWIDs from community involvement.

Limitations of the review

A major limitation of this scoping review was the limited number of articles that explored the impact of COVID-19 on SSPs in the U.S. during the study timeframe. Given that it has only been three years since the start of the pandemic, it is likely that the number of articles will grow in the coming years, and perhaps, further analyses regarding this topic can be conducted in the future. It is also important to note that most of these studies used interviews or surveys, both of which are prey to responder bias.

Regarding the method, first-tier secondary reviews of articles to include were conducted by a select group of four team members, and the second-tier review was conducted by two team members. Given the subjective nature of this review, it is likely that a larger number of authors designated to this task would have allowed for a less-biased list of included articles that adhered to this review’s goal. Similarly, the data charting process was completed by three authors, leading to a more subjective evaluation of the articles in this section. Additionally, the inclusion of quantitative, qualitative, and mixed-method studies made it difficult to summarize articles in the same fashion; while quantitative studies provided more objective findings, qualitative studies included SSP participant interviews and opinions, which were more subjective. Last, the topics of social isolation and loneliness, mental health changes, and fear of exposure to COVID-19 are rather personal and vulnerable topics, and participants may have been less apt to share their thoughts and experiences of this nature; therefore, data for such topics may be underestimated.

Implications for future research

This review highlighted the importance of staffing and supplies in the success of SSPs and how decreases in both drastically offset programs’ abilities to help PWID. Future studies should include a comparison of earlier staffing models to newer, post-pandemic staffing models to identify which changes have lasted and why programs have chosen to continue those changes. Additionally, a longer-term study of the impacts the pandemic had on individuals’ mental health and rehabilitation progresses could benefit how SSPs move forward in helping PWID recover from setbacks they experienced in the pandemic, as well as prepare SSPs to help PWID from similar potential situations in which they find themselves isolated from their supportive communities.

Overall, the findings of this review highlight the potential consequences of the COVID-19 pandemic on SSP operations, staff, and participants. Because of the increasing severity of drug use in the U.S., suboptimal SSP functions can have social and economic impacts on their communities. Thus, the results of this review should stimulate future investigations, as the outcomes affect many communities and are potential learning opportunities in the instances of future infectious disease outbreaks.

## Conclusions

SSPs have been crucial in curbing widespread addiction and the spread of diseases among PWID. Through these programs, individuals have increased access to clean needles and proper disposal mechanisms, as well as several other services. However, due to the emergence of the COVID-19 pandemic, many of these services were severely disrupted. The interruption led to decreased availability of these vital services, along with many other healthcare services across the world. Many SSPs experienced problems with supply, staffing, social isolation, and mental health. Examining how SSPs handled these issues could potentially provide useful solutions for preventing similar disruptions in the future. More studies are necessary to ensure a better understanding of how this pandemic has affected different sectors of healthcare services, as well as methods to improve and reduce the impact that such barriers may bring upon the individuals who are reliant on life-saving programs.
